# Functional Implications of MicroRNA-215 in TGF-β1-Induced Phenotypic Transition of Mesangial Cells by Targeting CTNNBIP1

**DOI:** 10.1371/journal.pone.0058622

**Published:** 2013-03-12

**Authors:** Jiao Mu, Qi Pang, Yan-Hong Guo, Ji-Gang Chen, Wei Zeng, Yong-Jun Huang, Jun Zhang, Bing Feng

**Affiliations:** Institute of Nephrology of Chongqing and Department of Nephrology, Xinqiao Hospital, Third Military Medical University, Chongqing, People’s Republic of China; The Chinese University of Hong Kong, China

## Abstract

Mesangial cell (MC) phenotypic transition is crucial for the progression of diabetic nephropathy. A major stimulus mediating high glucose-induced MC phenotypic transition is TGF-β1. Our current study focuses on microRNA-215 (miR-215) and investigates its role in TGF-β1-mediated MC phenotypic transition. Using real-time quantitative PCR (qRT-PCR) and northern blotting, we determined that the miR-192/215 family is dramatically upregulated under diabetic conditions both in vitro and in vivo. Gain- and loss-of-function approaches demonstrated that miR-215 inhibition significantly inhibited TGF-β1-induced mouse mesangial cell (MMC) phenotypic transition, whereas miR-215 upregulation promoted MMC phenotypic transition. Interestingly, these changes were not detected in cells that were treated with TGF-β1 and miR-192 mimics or inhibitors. These results suggest that miR-215 participates in TGF-β1-induced MMC phenotypic transition. Luciferase reporter assays were used to identify whether catenin-beta interacting protein 1 (CTNNBIP1) is a direct target of miR-215, which was predicted by bioinformatic analysis. Mechanistic studies revealed that CTNNBIP1 suppresses Wnt/β-catenin signaling and that miR-215 promotes β-catenin activation and upregulates α-SMA and fibronectin expression in TGF-β1-treated MMCs by targeting CTNNBIP1. In addition, in vivo miR-215 silencing with a specific antagomir significantly increased CTNNBIP1 protein expression, resulting in reduced β-catenin activity and decreased α-SMA and fibronectin expression in db/db mouse kidney glomeruli. Taken together, our findings indicate that miR-215 plays an essential role in MC phenotypic transition by regulating the CTNNBIP1/β-catenin pathway, which is related to the pathogenesis of diabetic nephropathy.

## Introduction

Diabetic nephropathy (DN) is a major microvascular diabetic complication that is characterized by glomerular hypertrophy, extracellular matrix (ECM) accumulation, glomerulosclerosis, and ultimately, end-stage kidney disease (ESKD) [1]–[3]. Central to the pathophysiology of DN are mesangial cells (MCs), which undergo glomerular injury-induced programmed myofibroblast transdifferentiation, also termed ‘activation’ or ‘phenotypic transition,’ into myofibroblasts under diabetic conditions [4]–[6]. Increasing evidence [7]–[10] indicates that hyperglycemia initiates MC phenotypic transition. Several cytokines and growth factors have been implicated in the effects of high glucose on MCs; the foremost among these is the profibrotic growth factor TGF-β1. Moreover, numerous reports [11]–[13] have demonstrated that high glucose increases TGF-β1 levels in glomerular MCs, whereas neutralizing anti-TGF-β1 antibodies prevent these changes [14], suggesting that TGF-β1 is necessary for regulating high glucose-induced MC activation. However, the mechanisms by which TGF-β1 induces MC phenotypic changes during the diabetic nephropathy progressive fibrosis phase are not fully defined.

MicroRNAs (miRNAs) are endogenous non-coding small RNAs that modulate gene expression by binding to the 3’-untranslated regions (3’-UTRs) of target mRNAs, which degrades mRNA or blocks protein translation at the post-transcriptional level [15]-[18]. Recently, miRNA expression profiles [19] revealed that several miRNAs (miR-192, miR-194, miR-204, miR-215, and miR-216) are highly and nearly exclusively expressed in the kidney. Furthermore, dysregulation of these key miRNAs is closely linked to progressive renal glomerular and tubulointerstitial fibrosis, which is correlated with reduced renal function [20]–[22]. New findings revealed that the miR-192/215 family regulates E-cadherin expression and mediates the TGF-β1/CTGF-induced epithelial to mesenchymal transition (EMT) in proximal tubular epithelial cells by downregulating ZEB2 expression. However, miR-192/215 expression in MCs did not affect matrix protein expression in the absence or presence of TGF-β1 [21]. Thus, further functional studies of miR-192/215 are essential to unravel the molecular mechanisms underpinning DN-associated renal fibrosis.

In this study, we analyzed the mechanisms of diabetes-induced MC activation. We found that miR-215 is a positive regulator of Wnt/β-catenin signaling, which appears to be critical in TGF-β1-induced MC phenotypic transition specifically by suppressing CTNNBIP1. In addition, in vivo miR-215 knockdown with antagomir-215 normalized CTNNBIP expression and inhibited Wnt/β-catenin signaling and expression of downstream genes α-SMA and fibronectin in the db/db mouse kidney. Taken together, these results demonstrate a significant role for miR-215 in diabetic kidney disease.

## Materials and Methods

### Cell culture and treatment

Primary MMCs were isolated and cultured as described previously [23] and were cultured in RPMI-1640 medium supplemented with 10% fetal bovine serum (FBS). The normal control group media contained 5.6 mM glucose. To explore the effect of high glucose on MMCs, either 24.4 mM glucose or 24.4 mM mannitol was added to the high glucose media (30 mM final concentration) or mannitol control, respectively [24]. Cells were incubated in a humidified incubator at 37°C with 5% CO_2_. TGF-β1 treatment was performed by culturing cells in media containing 2% FBS and 10 ng/ml recombinant human TGF-β1 (PeproTech, Rocky Hill, NJ) for the indicated times following previously described protocols [20]-[21].

### Real-time quantitative PCR (qRT-PCR)

Total RNA, including microRNA, was extracted from cultured cells and tissues using TRIzol reagent (Invitrogen, Carlsbad, CA) according to the manufacturer’s instructions. miRNA qRT-PCR was performed using a TaqMan MicroRNA Assay (Applied Biosystems, Foster City, CA) and an Applied Biosystems 7500 Fast real-time PCR system, as reported previously[25]. U6 snRNA (Applied Biosystems) was used as an endogenous normalization control. The PCR cycling conditions were as follows: 95°C for 10 min, followed by 40 cycles of 95°C for 15 s and 60°C for 35 s. qRT-PCR was performed using SYBR Premix ExTaq^TM^ (Takara, Tokyo, Japan) according to the manufacturer’s instructions. GAPDH was used as an endogenous normalization control. The PCR primer sequences were as follows: GAPDH (forward 5’-GACATCAAGAAGGTGGTGAAGC-3’; reverse 5’-GAAGGTGGAAGAGTTGGGAGTT-3’), CTNNBIP1 (forward 5’-AGGAAGATGGGGTCAAACCTG-3’; reverse 5’-CATCACCACGTCCTCTGCAC-3’). Primer and probe details for the other genes have been previously reported [26]–[28]. Individual samples were run in triplicate, and each experiment was repeated at least 3 times. Data analyses were performed using the comparative CT (ΔΔCT) method for calculating relative gene expression.

### Northern blot

Northern blots were performed as described previously [29] using an miR-192 probe (5’-CTGACCTATGAATTGACAGCC-3’) and an miR-215 probe (5’-GTCTGTCAAATCATAGGTCAT-3’). U6 snRNA (5’-ATATGGAACGCTTCACGAATT-3’) was used as an endogenous normalization control. All of the probes were γ-^32^P-ATP-labeled at the 5’ end and were purchased from Exiqon (Woburn, MA, USA).

### Computational target prediction and functional annotation

MiRNA target prediction was performed as described by Abdimajid et al. [30]. Briefly, the computational target prediction tools TargetScan (http://www.targetscan.org), PicTar (http://pictar.mdc-berlin.de) and miRanda (http://www.microrna.org) were used to assess potential miR-192/215 targets sites, and the results were compared. Target gene annotations that were available in the Gene Ontology (GO), Universal Protein Resource (UniProt), and Kyoto Encyclopedia of Genes and Genomes (KEGG) databases were utilized to illustrate which functional pathways may be regulated by miR-192/215.

### Luciferase reporter constructs and assays

Luciferase reporter constructs were used, and luciferase assays were performed as described previously [31]. Briefly, the mouse CTNNBIP1 3’-UTR sequence was amplified by PCR from mouse genomic DNA, digested with SpeI and HindIII restriction enzymes (Promega, Madison, WI), and ligated into the pMIR-REPORT luciferase vector multiple cloning site (Ambion, Austin, TX) to yield pMIR-CTNNBIP1 3′-UTR (CTNNBIP1 3′-UTR). Another pMIR-REPORT luciferase construct containing the CTNNBIP1 mRNA 3′-UTR with a mutation in the putative miR-192/215 seed region (UAGGUCA to UACCGCA) was generated as a negative control and named Mut-CTNNBIP1 3′-UTR. CTNNBIP1 3′-UTR primers were as follows: forward 5′-GCGCACTAGTTCTTGACCAACGGAAAC-3′; reverse 5′-GCGCAAGCTTACCTACAGCCAATCAAAG-3′. Mutagenesis primers for the miR-192/215 target site in the CTNNBIP1 3′-UTR were as follows: forward 5′-GCGCACTAGTTCTTGACCAACGGAAAC-3′; reverse 5′-GCGCAAGCTTCACATTCCTAAAAATGCGGTA-3′. MMCs were plated in 6-well plates and allowed to reach 60%-80% confluence overnight. Cells were then co-transfected with a reporter construct (pMIR-null REPORT plasmid, pMIR-CTNNBIP1 3′-UTR, pMIR-CTNNBIP1 3′-UTR-Mut) and miR-215 mimic, miR-192 mimic or miR-control. After 24 hours, cells were harvested, and luciferase activity was measured using the Dual-Luciferase Reporter Assay System (Promega) according to the manufacturer’s recommendations. Luciferase activity was normalized to control TK Renilla construct expression (pRL-TK, Promega) as previously reported [32].

### Transient transfection

All of the transient transfections were performed with Lipofectamine 2000 Reagent (Invitrogen). For gain- and loss-of-function studies, miRNA oligonucleotide transfections were performed according to an established protocol [33], [34]. Briefly, exponentially growing MMCs were seeded in 6-well plates at a density of 2×10^5^ cells per well and were grown overnight to 60%-80% confluency. Next, an miRNA mimic (Pre-miR^TM^ miRNA precursor), an miRNA inhibitor (Anti-miR^TM^ miRNA inhibitor), or a matched miRNA control (non-specific miRNA) (Ambion) was added to the culture media at a final concentration of 100 nM according to the manufacturer’s recommendations. Transfection efficiency (>90%) was measured by qRT-PCR. For small interfering RNA (siRNA) inhibition studies, CTNNBIP1 and β-catenin siRNA were purchased from Santa Cruz Biotechnology (Santa Cruz, CA) and were introduced into the MMCs at final concentrations of 30 and 100 nM, respectively, according to the siRNA transfection protocol. Control siRNA-transfected MMCs (the control siRNA sequence will not degrade CTNNBIP1 or β-catenin) were used as the negative control. Transfection efficiency (>80%) was measured by qRT-PCR. After 6 hours of transfection, the medium was replaced with normal glucose or low-serum (2% FBS) medium containing TGF-β1, and cells were incubated for the indicated times. For CTNNBIP1 overexpression studies, pcDNA3.1-CTNNBIP1 expression plasmids were constructed and transfected as previously reported [35], and construct accuracy was confirmed by DNA sequencing.

### Western blot

Western blotting was performed as described previously [36]–[39]. The following antibodies were used: anti-CTNNBIP1 antibody (1∶200; Santa Cruz), anti-active β-catenin antibody (1∶1000, Millipore, Billerica, MA), anti-total β-catenin antibody (1∶1000, BD Biosciences, Franklin Lakes, NJ), anti-α-SMA antibody (1∶200; Sigma, St. Louis, MO), and anti-fibronectin antibody (1∶200; Santa Cruz); anti-β-actin antibody (1∶1000; Sigma) was used as a loading control.

### Immunohistochemistry and Immunocytochemistry

Immunohistochemical staining was performed on tissues as described previously [40]. The following antibodies were used: anti-CTNNBIP1 antibody (1∶200; Santa Cruz) and anti-α-SMA antibody (1∶200; Sigma). Quantitative analyses of CTNNBIP1-positive renal glomerular staining were performed following previously described protocols [40]. Mesangial cells were examined by immunocytochemical staining of α-SMA with anti-α-SMA antibodies (1∶200; Sigma) using a previously reported method [41].

### Enzyme-linked immunosorbent assay (ELISA)

Fibronectin protein expression was quantified using an ELISA kit (Assaypro, Winfield, MO) according to the manufacturer’s instructions. ELISA results were normalized to total protein concentrations that were measured with the BioRad Protein Assay reagent. The results were expressed as micrograms per milligram of total protein.

### Animal studies

Male 4-week-old C57BL/KsJ type 2 diabetic db/db mice and nondiabetic db/m littermates were originally purchased from Jackson Laboratories (Bar Harbor, ME, USA). Mice were given ad libitum access to food and water and maintained at 23 ± 1°C room temperature with 55 ± 5% humidity and a 12-hour light/dark cycle. Antagomirs were designed and synthesized as described previously [42]. The antagomir-215 sequence was 5’-g_s_u_s_cugucaaaucauaggu_s_c_s_a_s_g_s_-Chol-3’, and the antagomir-control sequence was 5’-g_s_a_s_cagugaauucuuugcu_s_g_s_u_s_g_s_-Chol-3’ (lower case letters were 2’-O-methyl-modified nucleotides; subscripts indicated a phosphorothioate linkage; Chol represents cholesterol linked through a hydroxyprolinol linkage). Each oligonucleotide was purified by high-performance liquid chromatography (HPLC; Sigma) and dissolved in PBS. Db/db mice that were 8 weeks old were treated with 80 mg/kg antagomir-215 (n = 6) or antagomir-control (n = 6) via the tail vein for three consecutive days [42], [43]. The mice tolerated the antagomirs well and did not exhibit signs of discomfort. Body weight, blood glucose levels, and urinary albumin excretion were monitored weekly (data not shown). The mice were sacrificed three weeks after the last injection, and the kidneys were collected from the mice as described previously [44]. One piece of kidney tissue was formalin-fixed for immunohistochemistry, and the remaining cortices were stored at −70°C until use. All of the experiments were conducted in compliance with the National Institutes of Health Guide for the Care and Use of Laboratory Animals and with approval of the Third Military Medical University Animal Experiments Ethics Committee.

### Statistical analyses

All of the data are presented as the means ± SE. Statistical analyses were performed using Student’s t-test to evaluate the differences between two paired groups or a one-way analysis of variance (ANOVA) followed by LSD tests to assess differences for multiple comparisons among groups. A *P* value less than 0.05 was considered to be statistically significant. The data were analyzed with Statistical Product and Service Solutions 13.0 (SPSS 13.0) software.

## Results

### MiR-192/215 expression is upregulated under diabetic conditions both in vitro and in vivo

**Figure 1:**
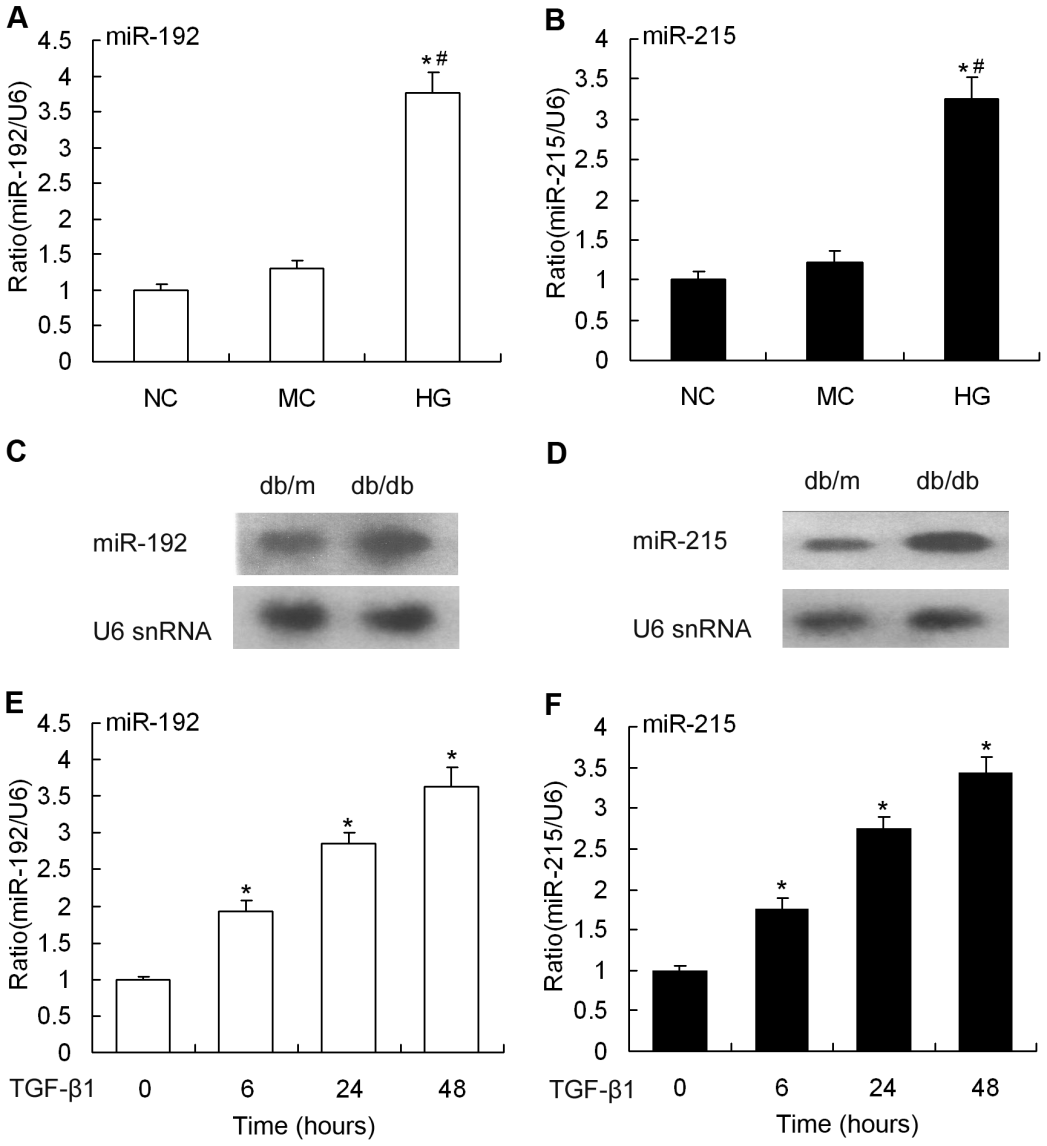
miR-192/215 expression upon treatment with high glucose and TGF-β1. (A and B) MMCs were treated with high glucose (30 mM) for 48 hours, and miR-192/215 expression was determined by qRT-PCR, normalized to U6 snRNA. Data are shown as means ± SE (n = 3 per group) (*P<0.05 compared with normal control, *^#^P*<0.05 compared with mannitol control). (C and D) miR-192/215 expression in glomeruli from mouse models of type 2 diabetes (db/db mice, 10 weeks of age) was detected by northern blot and normalized to U6 snRNA. Data are shown as means ± SE (n = 6 per group). (E and F) qRT-PCR analysis of miR-192/215 expression in MMCs treated with TGF-β1 (10 ng/ml) at different time points (0, 6, 24, and 48 hour).U6 snRNA serves as a loading control. Data are shown as means ± SE (n = 3 per group) (*P<0.05 compared with normal control). NC, normal control; MC, mannitol control; HG, high glucose.

To determine the potential functions of miR-192/215 in DN pathogenesis, we first analyzed changes in miR-192/215 expression by qRT-PCR and northern blotting. We tested MMCs that were exposed to high glucose in vitro and renal glomeruli from db/db mice in vivo. As shown in Figs. A and B, there was a significant increase in both miR-192 and miR-215 expression in MMCs that were treated with 30 mM glucose for 48 hours. Similarly, these two miRNAs were highly expressed in 10-week-old db/db mice, which is when the animals develop obvious biochemical and histological changes that are consistent with DN (Figs. 1C and D). These results are consistent with a previous report [20]. Current reports [11]–[13], [45] indicate that DN pathogenesis is attributable to elevated glucose concentrations, which act through the TGF-β1 system. Thus, we also assessed the effect of TGF-β1 on miR-192/215 expression in cultured MMCs. We observed a striking induction of miR-192 6 hours after stimulation; the levels gradually increased and peaked 48 hours after stimulation. MiR-215 expression in MMCs after TGF-β1 stimulation displayed a similar trend (Figs. 1E and F). These data convincingly show that miR-192/215 is highly expressed under diabetic conditions. Furthermore, TGF-β1 induced miR-192/215 expression increases in DN.

### CTNNBIP1 is a potential miR-215/192 target

**Figure 2:**
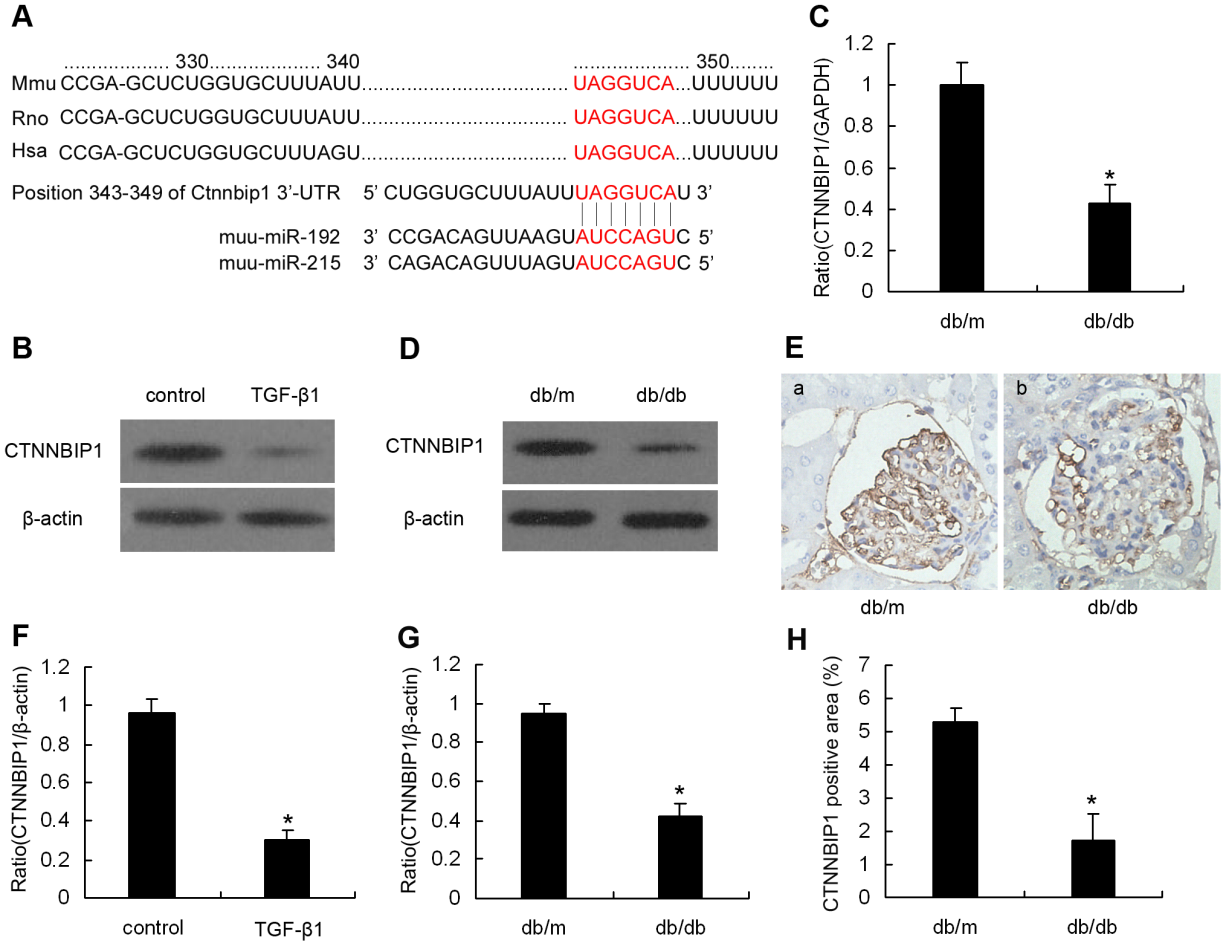
CTNNBIP1 is a potential miR-192/215 target. (A) Sequence alignment of miR-192/215 and predicted binding sites in the 3’-UTR of CTNNBIP1 (http://www.targetscan.org). (B) Protein expression levels of CTNNBIP1 in MMCs in response to TGF-β1 (10 ng/ml for 48 hours). (F) is the quantification of the results shown in (B).Data are shown as means ± SE (n = 3 per group) (*P<0.05 compared with the control group).CTNNBIP1 mRNA (C) and protein (D and E) levels in glomeruli from type 2 diabetic mice (db/db mice, 10 weeks of age). (G) is the quantification of the results shown in (D). (H) Quantification of renal glomerular CTNNBIP1 positive staining area shown in (E). Data are shown as means ± SE (n = 6 per group) (*P<0.05 compared with control db/m mice). Magnification, ×400 in E.

To examine the role of miR-192/215 in DN, we focused our attention on elucidating the miR-192/215 target mRNA. Computational analysis indicated that CTNNBIP1 is a potential miR-192/215 target because its 3’-UTR is perfectly matched to the miR-192/215 seed region (Fig. 2A). Target gene annotations that were available in the Uniprot and KEGG databases revealed that CTNNBIP1 is a novel β-catenin-interacting protein, which negatively regulates Wnt/β-catenin signaling by inhibiting the interaction between β-catenin and TCF/LEF family members. Emerging evidence suggests that altered Wnt/beta-catenin signaling is linked to renal fibrosis pathogenesis [27], [46], [47] Thus, we hypothesized that CTNNBIP1 may be the most statistically significant miR-192/215 target gene. Because TGF-β1 treatment increased miR-192/215 expression, CTNNBIP1 was likely to be suppressed after TGF-β1 treatment. To confirm this hypothesis, we incubated MMCs with TGF-β1 for 48 hours, and CTNNBIP1 protein levels were detected by western blotting. Fig. 2B demonstrates a significant reduction in CTNNBIP1 protein levels in TGF-β1-treated MMCs, consistent with our in vivo observations. A parallel decrease in CTNNBIP1 mRNA (Fig. 2C) and protein (Figs. 2D and E) expression in db/db mouse glomeruli was observed compared with the expression in db/m mice, suggesting that miR-192/215 expression negatively correlates with CTNNBIP1 levels in vitro and in vivo.

### CTNNBIP1 is a bona fide miR-215, but not miR-192 target

**Figure 3:**
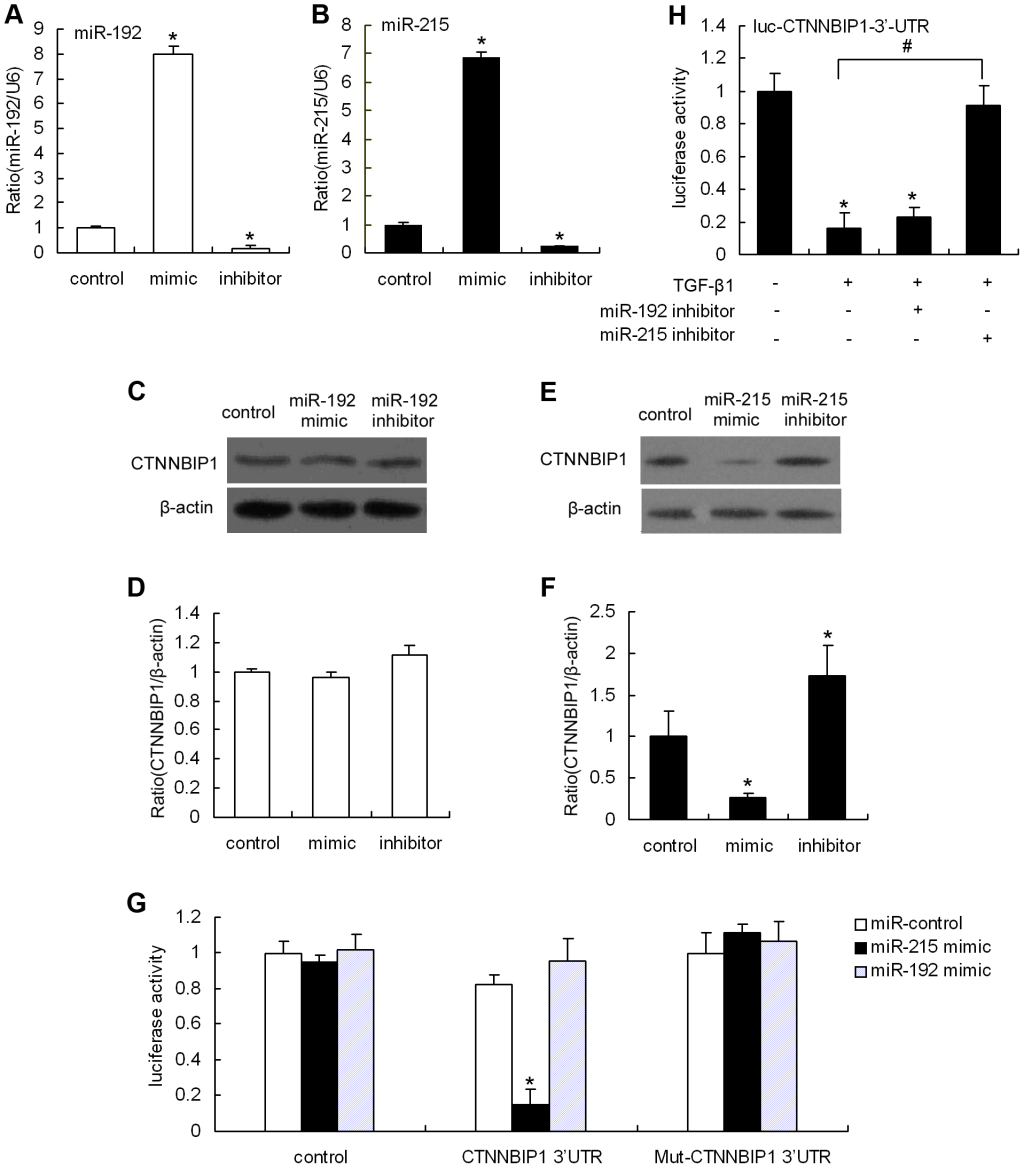
CTNNBIP1 is a direct target gene of miR-215. MMCs were treated with an miR-192 mimic (100 nM), an miR-192 inhibitor (100 nM) or the miR-control (100 nM) for 24 hours, miR-192 levels were determined by qRT-PCR (A), and subsequently analyzed for the level of CTNNBIP1 protein by Western blotting (C). (D) is the quantification of the results shown in (C). qRT-PCR results were normalized to U6 snRNA. Data are shown as means ± SE (n = 3 per group) (*P<0.05 compared with the miR-control group). MMCs were treated with an miR-215 mimic (100 nM), an miR-215 inhibitor (100 nM) or the miR-control (100 nM) for 24 hours, miR-215 levels were determined by qRT-PCR (B), and subsequently analyzed for the level of CTNNBIP1 protein by Western blotting (E). (F) is the quantification of the results shown in (E). qRT-PCR results were normalized to U6 snRNA. Data are shown as means ± SE (n = 3 per group) (*P<0.05 compared with the miR-control group). (G) Transient transfection of MMCs with the CTNNBIP1 3’-UTR or Mut-CTNNBIP1 3’-UTR constructs along with the miR-215 mimic, miR-192 mimic or miR-control, introduced 24 hours after transfection. Luciferase activities were measured and normalized to the control Renilla luciferase activity. Reporters without the CTNNBIP1 3’-UTR (control) or with Mut-CTNNBIP1 3’-UTR were used as the negative controls. Data are shown as means ± SE (n = 3 per group) (*P<0.05 compared with the cells transfected with the miR-control plus CTNNBIP1 3’-UTR). (H) For the TGF-β1 treatment experiments, cells were transfected with the luciferase reporter CTNNBIP1 3’-UTR alone or in combination with an miR-215 inhibitor (100 nM), an miR-192 inhibitor (100 nM) or an miR-control (100 nM), followed by treatment with TGF-β1 (10 ng/ml) for 24 hours. Luciferase activity was then analyzed (*P<0.05 compared with the untreated cells group, *^#^P* <0.05 as indicated).

To validate that CTNNBIP1 is indeed an miR-192/215 target, we first examined whether miRNA mimic or inhibitor transfection would affect CTNNBIP1 protein production. Transfection efficiency was measured by qRT-PCR (Figs. 3A and B). As shown in Figs. 3E and F, CTNNBIP1 protein expression was downregulated in MMCs that were transfected with the miR-215 mimic compared with the control group. In contrast, the miR-215 inhibitor markedly upregulated CTNNBIP1 protein levels. Interestingly, CTNNBIP1 protein was not altered in the miR-192 mimic- or inhibitor-treated MMCs compared with the control group (Figs. 3C and D). These results indicate that miR-215, but not miR-192, targets endogenous CTNNBIP1 in MMCs. Next, we further verified whether CTNNBIP1 is a direct miR-215 target using luciferase reporter assays. As shown in Fig. 3G, the miR-215 mimic significantly inhibited CTNNBIP1 3’-UTR luciferase activity by 90% relative to the negative miR-control but did not inhibit the two control constructs without the 3’-UTR (first pair of bars) or the Mut-CTNNBIP1 3’-UTR (third pair of bars). Conversely, the miR-192 mimic did not decrease luciferase activity of this same CTNNBIP1 3’-UTR construct. These results confirm that the CTNNBIP1 3’-UTR sequence is recognized by miR-215 and that CTNNBIP1 is an miR-215 target. Furthermore, we observed a marked decrease in CTNNBIP1 3’-UTR luciferase reporter activity in TGF-β1-treated MMCs compared with untreated cells. When TGF-β1-treated cells were transfected with the miR-215 inhibitor, the TGF-β1-induced decrease in luciferase activity in CTNNBIP1 3’-UTR reporter-transfected cells was abolished. However, the miR-192 inhibitor could not reverse these effects (Fig. 3H). These results further demonstrate that TGF-β1 downregulates CTNNBIP1 protein expression through an miR-215-regulated pathway.

### TGF-β1 induces MC phenotypic transition by regulating miR-215 expression

**Figure 4:**
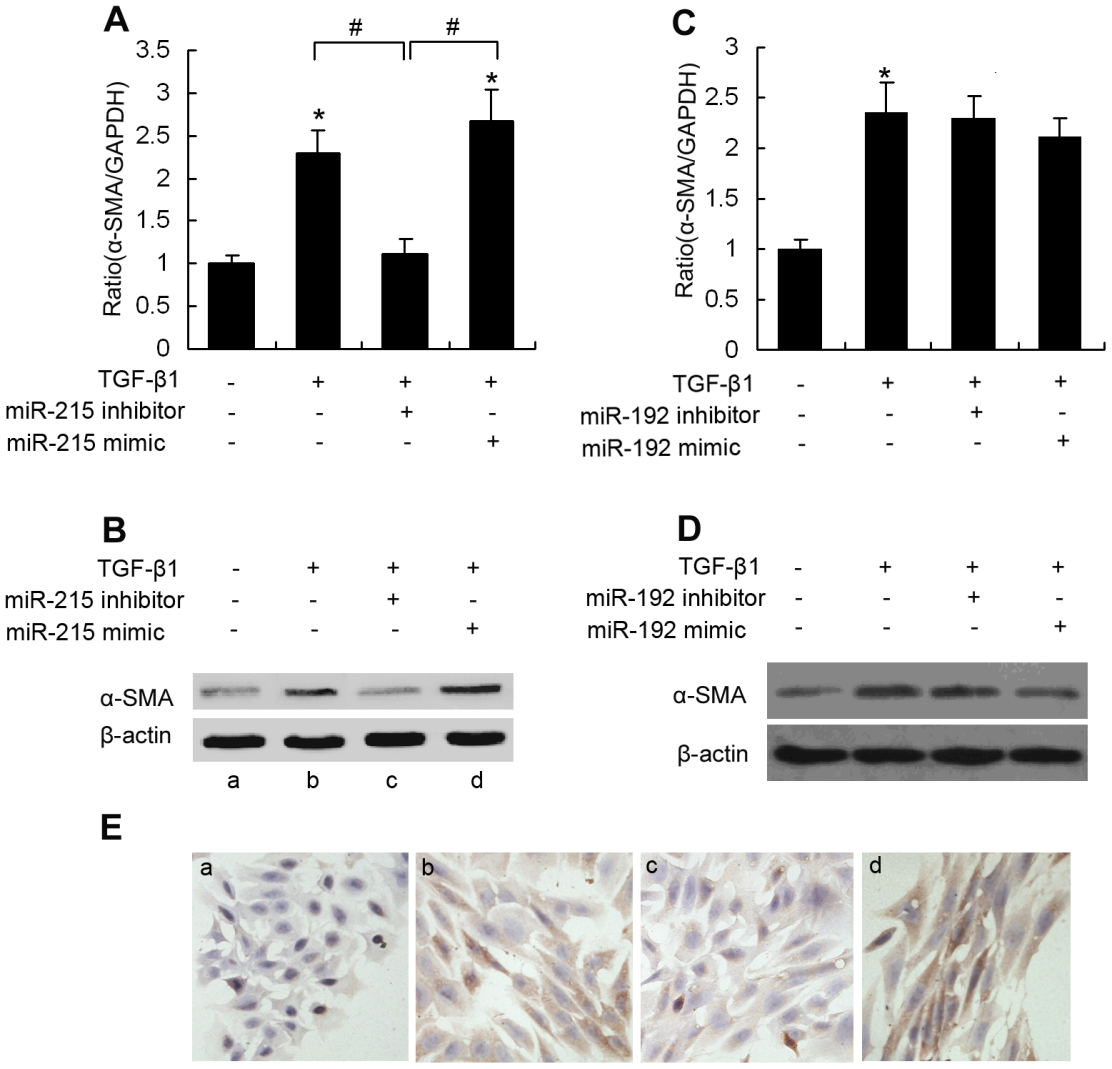
miR-215 regulates MMC phenotypic transition induced by TGF-β1. Cultured MMCs were transfected with an miR-215 mimic (100 nM), an miR-215 inhibitor (100 nM) or an miR-control (100 nM), followed by treatment with TGF-β1 (10 ng/ml) for 48 hours. qRT-PCR (A) and western blot (B)were used to analyze the expression of α-SMA levels in the cells. qRT-PCR results were normalized to GAPDH. Data are shown as means ± SE (n = 3 per group) (*P<0.05 compared with the untreated cells group, *^#^P*<0.05 as indicated). Cultured MMCs were transfected with an miR-192 mimic (100 nM), an miR-192 inhibitor (100 nM) or an miR-control (100 nM), followed by treatment with TGF-β1 (10 ng/ml) for 48 hours. qRT-PCR (C) and western blot (D)were used to analyze the expression of α-SMA levels in the cells. qRT-PCR results were normalized to GAPDH. Data are shown as means ± SE (n = 3 per group) (*P<0.05 compared with the untreated cells group, *^#^P*<0.05 as indicated). (E) Immunocytochemical staining for α-SMA in MMCs of different treatment groups. a, untreated MMCs; b-d, MMCs treated with TGF-β1 alone, TGF-β1 plus miR-215 inhibitor, or TGF-β1 plus miR-215 mimic, respectively. Magnification, ×200 in E.

To investigate the involvement of the miR-192/215 family in TGF-β1-induced MC phenotypic transition, MMCs were transfected with an miR-192 or an miR-215 inhibitor. TGF-β1 was added 6 hours after transfection. qRT-PCR, western blotting and immunocytochemistry were used to measure myofibroblast phenotypic marker α-SMA expression after TGF-β1 incubation for 48 hours, compared with miR-control-transfected cells. As expected, TGF-β1 treatment increased α-SMA expression at both the mRNA (Figs. 4A and C) and protein levels (Figs. 4B and D). However, this upregulation was significantly inhibited by pretreatment with a specific miR-215 inhibitor. In contrast, a specific miR-215 mimic enhanced TGF-β1-induced MMC changes (Fig. 4A and B). Interestingly, transfection with miR-192 mimic or miR-192 inhibitor did not alter TGF-β1-induced α-SMA expression in MMCs (Figs. 4C and D). Furthermore, MMCs displayed a typical spindle shape that is associated with increased α-SMA expression after TGF-β1 treatment; however, these phenotypic and morphological changes were reversed when cells were exposed to TGF-β1 and miR-215 inhibitor in combination for 24 hours (Fig. 4E).These data provide evidence that transdifferentiation from MC to myofibroblast can be induced by TGF-β1 and miR-215 participates in the MC phenotypic transition.

### CTNNBIP1 is a signal transducer that is involved in TGF-β1-induced MMC phenotypic transition and fibronectin expression

**Figure 5:**
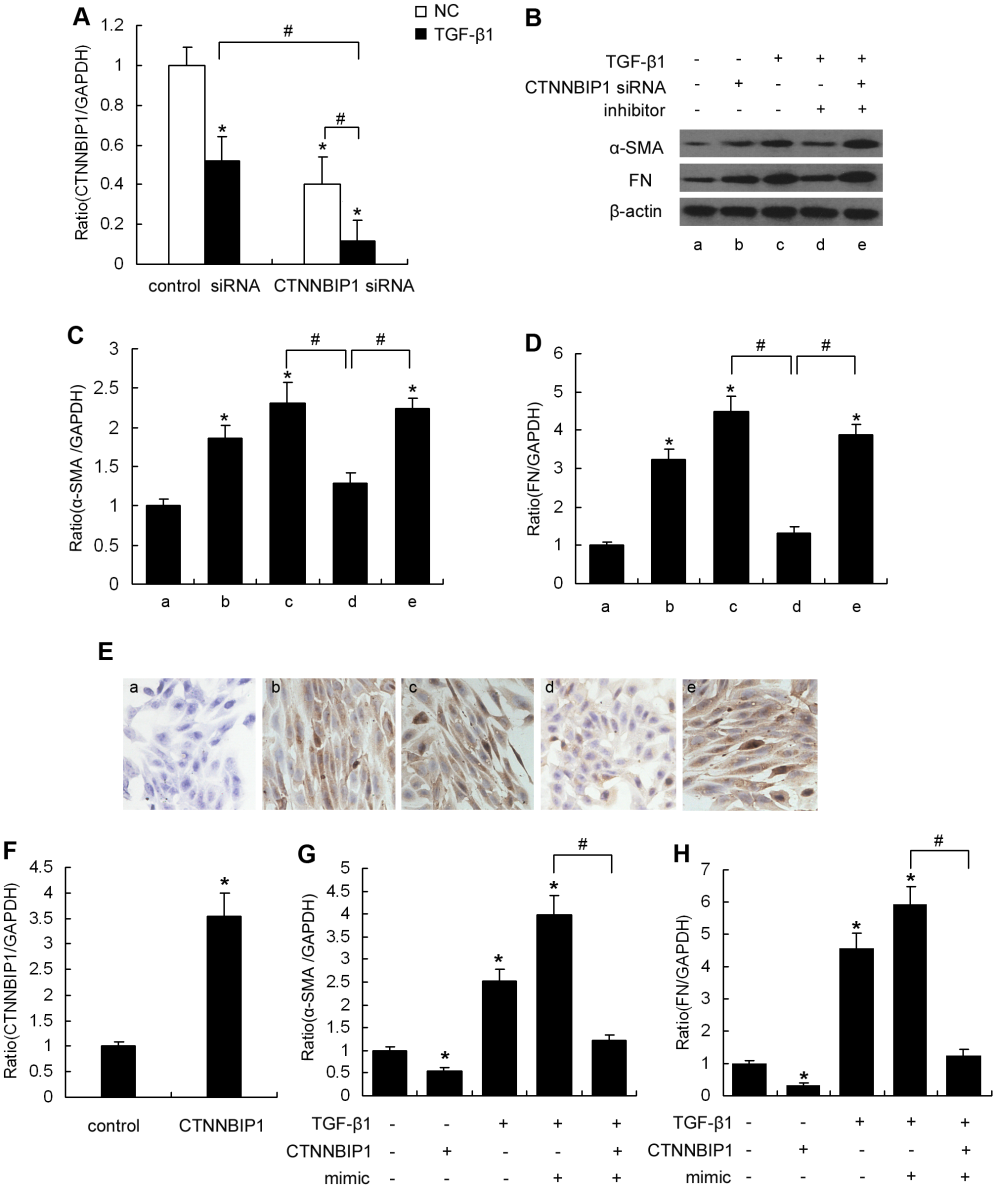
CTNNBIP1 is involved in miR-215-mediated MMC phenotypic transition. (A) MMCs were transfected with control siRNA (30 nM) or CTNNBIP1 siRNA (30 nM), and TGF-β1 (10 ng/ml) was added to indicated group at 12 hour after transfection. The expression of CTNNBIP1 was measured by qRT-PCR. Data are shown as means ± SE (n = 3 per group) (*P<0.05 compared with the normal control plus control siRNA group, *^#^P* <0.05 as indicated). MMCs were transfected with CTNNBIP1 siRNA (30 nM) and/or an miR-215 inhibitor (100 nM) as indicated. Following treatment with TGF-β1(10 ng/ml) or normal glucose for 48 hours, qRT-PCR and western blot were used to analyze the expression of α-SMA and FN mRNA (C and D) and protein (B) levels in the cells, respectively. qRT-PCR results were normalized to GAPDH. Data are shown as means ± SE (n = 3 per group) (*P<0.05 compared with the untreated cells group, *^#^P* <0.05 as indicated). (E) Immunocytochemical staining for α-SMA in MMCs of different treatment groups. a, untreated MMCs; b-e, MMCs treated with CTNNBIP1 siRNA, TGF-β1 alone, TGF-β1 plus miR-215 inhibitor, or TGF-β1 plus miR-215 inhibitor plus CTNNBIP1 siRNA, respectively. Magnification, ×200 in E. (F) MMCs were transfected with CTNNBIP1 expression plasmids (0.25 µg plasmid/well) for 24 hours, and qRT-PCR was used to analyze CTNNBIP1 expression levels. Control vectors were used as a negative control. qRT-PCR results were normalized to GAPDH. Data are shown as means ± SE (n = 3 per group) (*P<0.05 compared with the control vector group). MMCs were transfected with CTNNBIP1 expression plasmids (0.25 µg plasmid/well) or miR-215 mimics (100 nM) as indicated. TGF-β1 (10 ng/ml) was added to the indicated group 12 hours after transfection. α-SMA (G) and FN (H) expression levels were measured by qRT-PCR. Data are shown as means ± SE (n = 3 per group) (*P<0.05 compared with the control vector group, *^#^P* <0.05 as indicated). control, MMCs treated with control vectors; CTNNBIP1, MMCs treated with CTNNBIP1 expression vectors; mimic, miR-215 mimic; inhibitor, miR-215 inhibitor.

Our findings confirm that, in response to TGF-β1, miR-215 negatively regulates the CTNNBIP1 gene by targeting its 3’-UTR sequence. Therefore, we further examined the functional involvement of CTNNBIP1 in TGF-β1-induced miR-215-mediated MMC phenotypic transition. Specific CTNNBIP1 siRNA was used to determine CTNNBIP1 function. We observed that CTNNBIP1 expression was reduced by 60% and 80% in MMCs in the absence or presence of TGF-β1, respectively (Fig. 5A). Furthermore, we noticed that both TGF-β1 and CTNNBIP1 siRNA increased α-SMA and fibronectin expression in MMCs, whereas a specific miR-215 inhibitor significantly reduced this TGF-β1-induced effect. Importantly, when TGF-β1-treated MMCs were co-transfected with the miR-215 inhibitor in combination with CTNNBIP1 siRNA, the miR-215 inhibitor-mediated repression of α-SMA and fibronectin expression were largely abolished (Figs. 5B–E). To further examine whether CTNNBIP1 reduced MMC phenotypic transition under diabetic conditions, we overexpressed CTNNBIP1 using an expression plasmid. As shown in Fig. 5F, CTNNBIP1 expression was 3.5-fold higher in CTNNBIP1 plasmid-transduced MMCs compared with control plasmid-transduced cells. Moreover, qRT-PCR analysis demonstrated that CTNNBIP1 overexpression significantly decreased α-SMA and fibronectin levels in TGF-β1 and miR-215 mimic co-treated MMCs (Figs. 5G and H). These results indicate that the effect of miR-215 on TGF-β1-induced MMC phenotypic transition and fibronectin expression depends on CTNNBIP1.

### β-catenin is a signaling molecule downstream of CTNNBIP1 that is involved in miR-215-mediated MMC phenotypic transition and fibronectin expression

**Figure 6:**
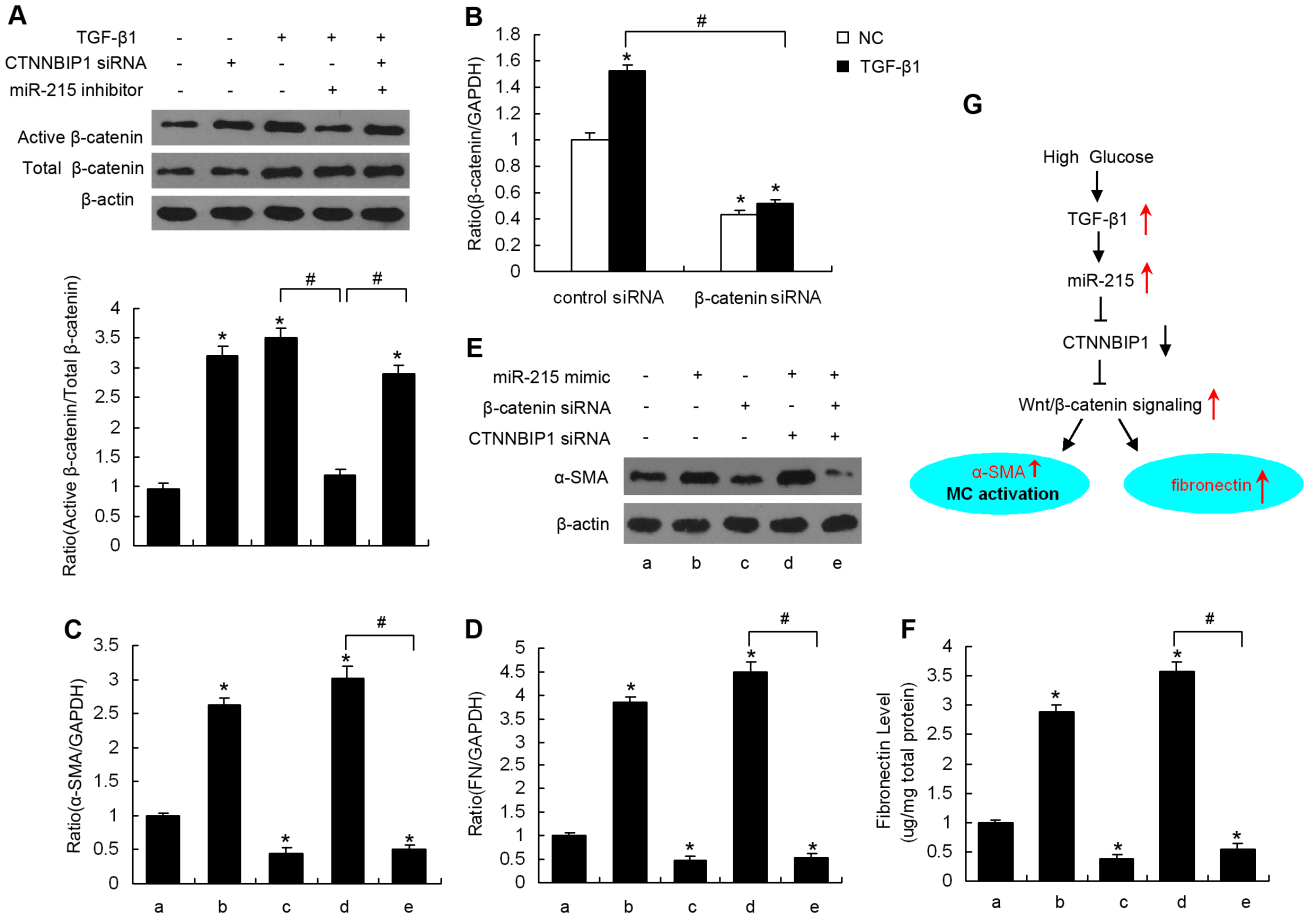
β-catenin is a signaling molecule downstream of CTNNBIP1 that is involved in miR-215-mediated phenotypic transition and fibronectin expression . MMCs were transfected with CTNNBIP1 siRNA (30 nM) and/or an miR-215 inhibitors (100 nM) as indicated. Following treatment with TGF-β1 (10 ng/ml) or normal glucose for 48 hours, western blot analyses were performed for quantitation of activated β-catenin and total β-catenin protein levels (A). Data are shown as means ± SE (n = 3 per group) (*P<0.05 compared with the untreated cells group, *^#^P*<0.05 as indicated). (B) MMCs were transfected with control siRNA (100 nM) or β-catenin siRNA (100 nM), and TGF-β1 (10 ng/ml) was added to the indicated group 12 hours after transfection. β-catenin expression was measured by qRT-PCR and was normalized to GAPDH. Data are shown as means ± SE (n = 3 per group) (*P<0.05 compared with the normal control plus control siRNA group, *^#^P* <0.05 as indicated). MMCs were transfected with an miR-215 mimics (100 nM) and/or CTNNBIP1 siRNA (30 nM) as indicated. Following β-catenin siRNA (100 nM) or control siRNA (100 nM) treatment for 48 hours, qRT-PCR was used to analyze the α-SMA (C) and FN (D) mRNA expression in the cells. qRT-PCR results were normalized to GAPDH. Cellular α-SMA protein levels were examined by western blotting (E). Media was harvested by centrifugation, and fibronectin in the supernatant was measured by ELISA (F). Fibronectin concentration was normalized to the total protein in the media. Data are shown as means ± SE (n = 3 per group) (*P<0.05 compared with the untreated cells group, *^#^P*<0.05 as indicated). (G) Schematic drawing demonstrating a previously uncharacterized mechanism for TGF-β1-induced MC phenotypic transition and fibronectin accumulation in DN. a, untreated MMCs; b–e, MMCs treated with miR-215 mimic, β-catenin siRNA alone, miR-215 mimic plus CTNNBIP1 siRNA, or miR-215 mimic plus CTNNBIP1 siRNA plus β-catenin siRNA, respectively.

Previous studies have indicated that CTNNBIP1 antagonizes Wnt/β-catenin signaling and its downstream genes by repressing constitutively activated β-catenin activity [37]. In this study, we addressed whether the miR-215/CTNNBIP1 pathway was involved in TGF-β1-induced MMC phenotypic transition by regulating β-catenin activity. Fig. 6A demonstrates that siRNA-mediated CTNNBIP1 knockdown resulted in a 3.3-fold increase in β-catenin activity under normal glucose conditions. Consistent with these results, α-SMA and fibronectin expression (Figs. 5B–E) also significantly increased compared with the control group, suggesting that CTNNBIP1 inhibits Wnt/β-catenin signaling in MMCs and further suppresses α-SMA and fibronectin expression. Because TGF-β1 treatment reduced CTNNBIP1 levels, we observed a 3.5-fold increase in β-catenin activity after treating MMCs with TGF-β1. Moreover, miR-215 inhibitor pretreatment significantly decreased the TGF-β1-mediated increase in activated β-catenin protein (Fig. 6A), α-SMA (Figs. 5B, C and E) and fibronectin (Figs. 5B and D) levels in MMCs. Most importantly, these changes were largely abolished when TGF-β1-treated MMCs were co-transfected with the miR-215 inhibitor in combination with CTNNBIP1 siRNA (Figs. 5B–E). These results indicate that miR-215 promotes β-catenin activation and upregulates α-SMA and fibronectin expression in TGF-β1-treated MMCs by targeting CTNNBIP1. To further identify whether β-catenin is a downstream effector of CTNNBIP1 that is involved in MMC phenotypic transition, we next knocked down β-catenin in MMCs with siRNA. We observed reduced β-catenin expression in MMCs by 57% and 66% in the absence or presence of TGF-β1, respectively (Fig. 6B). In addition, our data demonstrated that β-catenin mRNA expression was effectively reduced by β-catenin siRNA, which subsequently suppressed MMC α-SMA and fibronectin levels. These results confirm that α-SMA and fibronectin are Wnt/β-catenin signaling pathway target genes. Furthermore, when MMCs were co-transfected with the miR-215 mimic in combination with CTNNBIP1 siRNA, α-SMA and fibronectin expression were significantly increased relative to the control group. However, this upregulation was significantly inhibited by pretreatment with β-catenin siRNA (Figs. 6C–F). These results further suggest that in MMCs, CTNNBIP1 negatively regulates α-SMA and fibronectin expression by activating β-catenin, which may participate in miR-215-mediated phenotypic transition and TGF-β1-induced fibronectin expression (Fig. 6G).

### In vivo miR-215 knockdown reduces α-SMA and fibronectin expression in db/db mice

**Figure 7:**
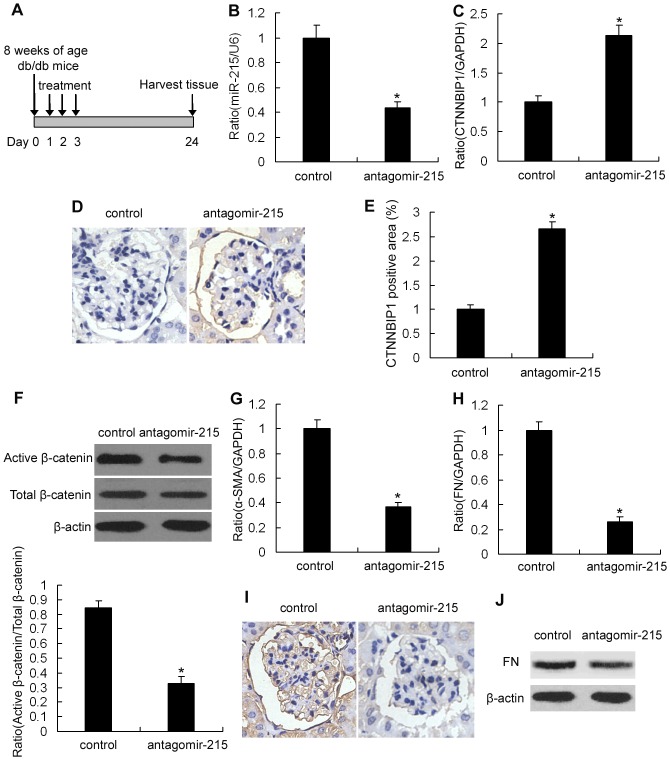
In vivo miR-215 inhibition reduces α-SMA and fibronectin expression. (A) Design of prevention study. Db/db mice (8 weeks of age) were treated with antagomir-control or antagomir-215 (80 mg /kg body weight, three injections in three consecutive days) via the tail vein, and the kidneys were harvested three weeks after the last injection. miR-215 expression was determined by qRT-PCR (B) and subsequently analyzed for CTNNBIP1 mRNA (C) and protein (D) expression levels in glomeruli from antagomir-215-treated mice by qRT-PCR and immunohistochemical staining, respectively. qRT-PCR results were normalized to U6 snRNA. Data are shown as means ± SE (n = 6 per group) (*P<0.05 compared with antagomir-control treated mice). (E) Quantification of renal glomerular CTNNBIP1 positive staining area shown in (D). (F) Western blot analyses and quantitation of the activated and total β-catenin protein levels. Data are shown as means ± SE (n = 6 per group) (*P<0.05 compared with antagomir-control treated mice). qRT-PCR was used to analyze α-SMA (G) and FN (H) mRNA expression levels in glomeruli of the antagomir-215-treated mice. qRT-PCR results were normalized to GAPDH. Data are shown as means ± SE (n = 6 per group) (*P<0.05 compared with antagomir-control treated mice). α-SMA (I) and FN (J) protein expression levels were detected in the glomeruli by immunohistochemical staining and western blotting, respectively. control, db/db mice treated with antagomir-control, antagomir-215, db/db mice treated with antagomir-215. Magnification, ×400 in H.

After establishing that miR-215 regulated α-SMA and fibronectin expression in MMCs via a CTNNBIP1/β-catenin-dependent pathway, we expanded our study to investigate whether these mechanisms were important in db/db mice. Antagomirs are efficient and specific endogenous miRNA silencers that are successful at reducing miRNA expression in most normal murine tissues for as long as three weeks after systemic treatment [42], [48]. Thus, we used antagomir-215 to knock down miR-215 expression in db/db mice and further examine the effects of miR-215 inhibition on the CTNNBIP1/β-catenin pathway (Fig. 7A). qRT-PCR analysis demonstrated that miR-215 expression was knocked down by 55% in the kidney cortices of the antagomir-215-treated mice compared with those in the control mice (Fig. 7B). To evaluate the in vivo relevance of miR-215 knockdown to CTNNBIP1 expression, we analyzed CTNNBIP1 expression changes by qRT-PCR and immunohistochemical staining. As shown in Figs. 7C, D and E, glomerular CTNNBIP1 expression in antagomir-215-treated mice was markedly increased at both the mRNA and protein levels compared with the control mice, suggesting that miR-215 regulates CTNNBIP1 expression in vivo. Moreover, β-catenin activity was significantly reduced because the antagomir-215 treatment increased CTNNBIP1 expression (Fig. 7F). Most importantly, a parallel decrease in α-SMA (Figs. 7G and I) and fibronectin (Figs. 7H and J) expression in antagomir-215-treated mouse renal glomeruli was observed compared with control mice. These results are consistent with our observations in vitro and suggest that miR-215 directly targets endogenous CTNNBIP1, which inhibits Wnt/β-catenin signaling and its downstream genes α-SMA and fibronectin in db/db mice in vivo.

## Discussion

In this study, we demonstrated for the first time that miR-215 plays a positive regulatory role in MC activation, which is associated with DN pathology. Most importantly, we provided evidence, both in vitro and in vivo, that miR-215 is a central component of MC transdifferentiation pathways that contribute to DN pathology by targeting CTNNBIP1 and promoting Wnt/β-catenin signaling.

Recent attention has been focused on miRNAs as principal mediators of fibrosis in diabetic renal disease [20], [21], [49]-[53], with a special emphasis on cross talk between specific renal miRNAs and TGF-β1-driven signaling [54]. TGF-β1 positively regulates miR-192 [20] and miR-216a [22] but negatively regulates the miR-200 family [52], resulting in enhanced ECM protein accumulation, which contributes to DN pathogenesis. Despite the growing number of studies that provide clear evidence linking miRNA dysregulation and prosclerotic factors to renal progressive fibrosis, few critical miRNAs have been identified. Thus, further research is necessary to elucidate the role of additional potential miRNAs in DN pathophysiology. Our previous study profiled miRNA expression in MCs that were exposed to hyperglycemic conditions; we observed significant upregulation of miR-192/215, which is exclusively expressed in human and mouse kidneys. Subsequently, northern blotting analysis confirmed that miR-192/215 expression was markedly higher in db/db mouse kidney glomeruli compared with db/m control mice. In this study, we further observed that TGF-β1 treatment upregulates miR-192/215 in a time-dependent manner from 0-48 h in MMCs. This was in contrast to a previous study [21] in which miR-192/215 was decreased by the same dose of TGF-β1 treatment in MMCs after 3 days. The differences in these time points may explain the discrepancy in TGF-β1-mediated changes in miR-192/215 expression [54]. Taken together, our findings strongly indicate that miR-192 and miR-215 were upregulated under diabetic conditions both in vitro and in vivo and that miR-192 and miR-215 may be critical regulators of TGF-β1 signaling, which accelerates the MC phenotypic transition.

Importantly, computational analysis predicted that CTNNBIP1 is an miR-192/215 family target gene. Gene annotations demonstrated that CTNNBIP1 prevented the interaction between β-catenin and TCF/LEF family members, thus acting as a negative regulator of the Wnt/β-catenin signaling pathway [35], [55]-[58]. The central effector of the canonical Wnt cascade is β-catenin, which binds to TCF or LEF to initiate Wnt target gene transcription, including the myofibroblast conversion marker α-SMA and the fibrotic matrix protein fibronectin [47], [59], [60]. Additionally, using both gain- and loss-of-function experiments, we determined that only miR-215 regulated endogenous CTNNBIP1 expression ex vivo. Interestingly, luciferase reporter assays further revealed that CTNNBIP1 is a direct miR-215 target in MMCs that are untreated or TGF-β1-treated. However, miR-192 mimics could not inhibit this same CTNNBIP1 3’-UTR luciferase reporter construct activity. These results indicated that miR-215, but not miR-192, recognizes the CTNNBIP1 gene 3’-UTR sequence and thereby decreases CTNNBIP1 expression. Our study thus highlights the importance of experimental validation of predicted miRNA targeting. Interestingly, we did not observe TGF-β1-induced changes in α-SMA expression in miR-192 mimic- or inhibitor-treated cells, consistent with a recent report by Wang et al. [21]. Their study revealed that miR-215 mimic transfection of MMCs had no effect on α-SMA expression in the absence or presence of TGF-β1. In the same study, we verified that miR-215 participates in TGF-β1-mediated MMC phenotypic transition. These contrasting findings may be due to the different cell models, experimental conditions, and miRNAs mimics/inhibitors that were used [54], [61], [62]. Thus far, the complex roles and regulation of the miR-192/215 family in MMCs remain unclear; thus, more studies are needed to clarify these aspects. Based on the above observations, we speculated that miR-215 promoted TGF-β1-induced MC injury in DN via its target gene CTNNBIP1 and the Wnt/β-catenin pathway.

To further investigate this hypothesis, we demonstrated that siRNA-mediated knockdown of CTNNBIP1 inhibited CTNNBIP1 production, which markedly increased β-catenin activity. Therefore, we confirmed that CTNNBIP1 is indeed an inhibitor of the Wnt/β-catenin signaling pathway by negatively regulating β-catenin activity in MMCs. Interestingly, we observed that CTNNBIP1 siRNA reversed the effects of an miR-215 inhibitor on the TGF-β1-mediated MMC phenotypic transition, indicating that CTNNBIP1 is involved in miR-215-mediated pro-differentiation effects in the TGF-β1-induced MMC phenotype transition. In support of this conclusion, it was recently reported that E2F1 plays a tumor suppressor role in colorectal cancer by activating CTNNBIP1 and inhibiting β-catenin activity [37]. MC activation, which is characterized by the induction of α-SMA expression, contributes to diabetic renal diseases [59]. Our results also demonstrate that transcriptionally active β-catenin induces α-SMA expression, which is indicative of MC activation and is accompanied by increased fibronectin expression in MMCs that are transfected with CTNNBIP1 siRNA and/or an miR-215 mimic. However, this upregulation was significantly inhibited by pretreatment with β-catenin siRNA. Indeed, accumulating evidence indicates that the Wnt/β-catenin signaling pathway is essential for human lung fibroblast [63] and HSC-T6 [64] cell activation by inducing α-SMA expression. This activation enhances collagen production and contributes to idiopathic pulmonary fibrosis and liver fibrogenesis. These observations led us to conclude that the miR-215/CTNNBIP1 pathway acts downstream of TGF-β1 signaling and functionally mediates MC activation and fibronectin expression by regulating the Wnt/β-catenin signaling pathway in diabetes. Importantly, our in vivo study demonstrated that antagonizing miR-215 expression in db/db mice significantly enhances endogenous CTNNBIP1 protein expression, resulting in reduced Wnt/β-catenin signaling and expression of downstream genes α-SMA and fibronectin, which is in agreement with the in vitro data. In support of these findings, a study by Long et al. [29] demonstrated that db/db mice that were injected with antagomirs targeting miR-29c ameliorated DN progression. Moreover, Thum et al. [43] reported that miR-21 silencing with a specific antagomir prevented cardiac dysfunction in a mouse model of heart failure. These results provide evidence for the therapeutic efficacy of antagomirs in diabetes. In addition, although we found a dramatic reduction in β-catenin activity that was accompanied by a parallel decrease in α-SMA and fibronectin expression in the kidney of antagomir-215 treated db/db mice, we will provide more convincing evidence that this effect is mediated through CTNNBIP1 by using both gain- and loss-of-function approaches in our future studies. Taken together, our findings demonstrate that miR-215 plays an essential role in diabetic nephropathy pathogenesis by regulating the CTNNBIP1/β-catenin pathway and further suggest that miR-215 is a potential therapeutic target for diabetic nephropathy.

In conclusion, our results demonstrate that miR-215 is a key endogenous gene-silencing factor that mediates TGF-β1-induced MC activation and fibronectin expression via a CTNNBIP1/β-catenin pathway. Furthermore, experiments using specific antagomir treatment in db/db mice provide evidence for the functional importance of miR-215 in diabetic nephropathy pathogenesis in vivo. The development of miR-215-based therapeutic strategies may attenuate or even reverse kidney fibrosis and dysfunction. This area represents a fascinating future opportunity for treating patients with diabetic kidney disease.
